# Postoperative discal pseudocyst after percutaneous endoscopic transforaminal discectomy treated by drainage: Case report

**DOI:** 10.1097/MD.0000000000030204

**Published:** 2022-08-26

**Authors:** Shuai Wang, Yang Yang, Xiuchun Yu, Zhengqi Chang

**Affiliations:** a Department of Orthopedics, 960th Hospital of PLA, Jinan 250031, Shandong, PR China; b The First Clinical Medical College, Shandong University of Traditional Chinese Medicine, Jinan 250014, Shandong, PR China.

**Keywords:** drainage, percutaneous endoscopic transforaminal lumbar discectomy, postoperative discal pseudocysts

## Abstract

This article reports 2 cases of symptomatic postoperative discal pseudocysts (PDP), a rare complication of percutaneous endoscopic transforaminal lumbar discectomy (PELD). In this report, we propose a possible mechanism of PDP and introduce an effective therapeutic strategy. To our knowledge, there have been no reports of the use of indwelling drainage techniques for the PDP treatment after PELD.

**Patient concerns:**

Herein, we report 2 cases of PDP after PELD in our hospital. Both patients had disc herniation at the L4/5 level, and the symptoms of low back pain and radiculopathy were significantly relieved after PELD. However, the signs in both 2 cases recurred 20 days after surgery. MRI indicated PDP in both 2 patients with high intensity on T1- and T2-weighted imaging in the primary surgical area.

**Interventions:**

Given the progressive symptoms in both cases, PELD was performed again and 3-lumen drainage catheters were placed at the surgical site for adequate drainage.

**Outcomes:**

The patient’s symptoms were significantly relieved after adequate drainage and disappeared 3 months after surgery. There was no clinical or MRI recurrence at the 6-month follow-up.

**Conclusion:**

According to operative findings, we found that PDP symptoms may not be attributable mainly to cyst compression but to the excessive accumulation of local inflammatory factors. Treatment of PELD combined with indwelling drainage is feasible and effective in treating PDP.

## 1. Introduction

Percutaneous endoscopic lumbar discectomy (PELD) is rapidly gaining popularity worldwide owing to its advantages, such as minor trauma, short operation time, safe and controllable operation, and quick recovery. However, this technique is inevitably accompanied with complications.^[1]^ In recent years, postoperative discal pseudocysts (PDP), a rare complication of PELD, have been reported in several cases. PDP mainly occurs in the short term after the operation, primarily manifesting as low back pain and radiating pain in the lower extremity and other symptoms similar to lumbar disc herniation.

In these 2 PDP reports, we demonstrated a novel strategy for PDP treatment, PELD combined with indwelling drainage, with satisfactory short-term efficacy. In addition, based on systematically reviewing the related literature on PDP and summarizing the clinical data of this group of patients, we propose new possible pathogenesis of PDP. We also presented the strategy of PELD combined with indwelling drainage to be feasible and effective. This report provides a reference for the prevention and treatment of PDP.

## 2. Case presentation

**Figure 1.**
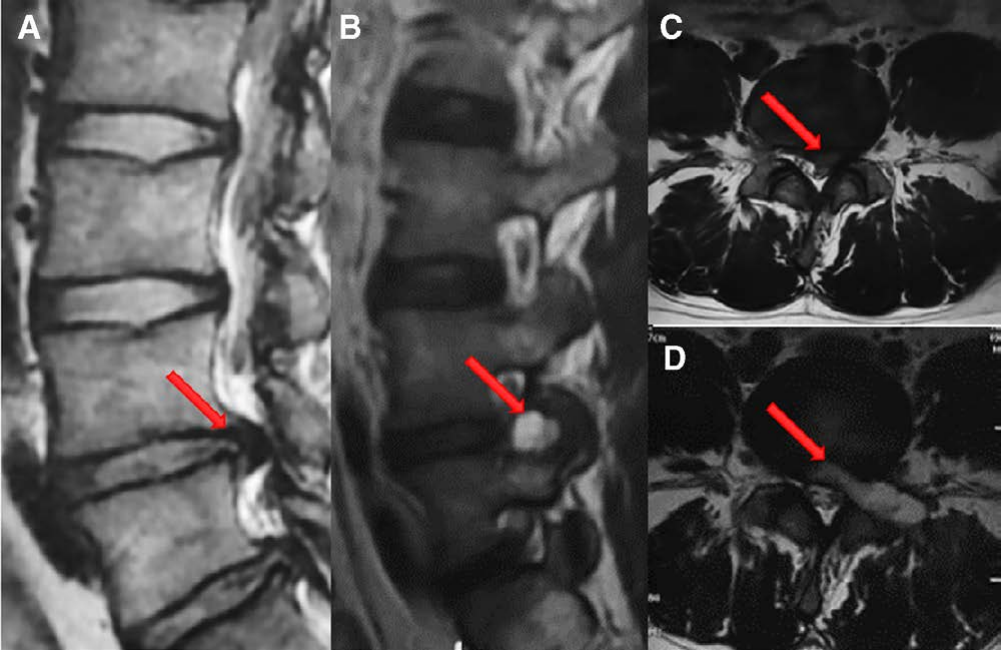
MRI images of Case 1 at the first and second admissions. (A, C) MRI of case 1 on the first admission showed that the L4/5 intervertebral disc herniated posteriorly, compressing the dural sac. (A) T2-weighted sagittal image. (C) T2-weighted cross-sectional image. (B, D) MRI of case 1 on the second admission showed an enhanced signal in T2-weighted images at the L4/5 level. (B) T2-weighted sagittal image. (D) T2-weighted cross-sectional image.

Case 1: A 24-year-old male presented with chronic low back pain for 1 year, aggravated and accompanied by radiating pain and numbness in the left lower extremity for half a year. Specialist physical examination revealed lameness and limited flexion and extension of the lumbar spine. At the same time, there was tenderness and percussion pain in the spinous process and between the spinous processes of the lower lumbar vertebrae and radiated to the left buttock and left lower extremity. The VAS score was 6 points. Right lower extremity muscle strength and sensation were normal. The skin of the left calf showed superficial hypesthesia on the outer side. The muscle strength of the left extensor dorsi muscle was grade 4, and the muscle strength of the rest of the left lower limb was normal. The left straight leg raise test was positive at 30°. At the same time, the left Achilles tendon reflex was weakened, but bilateral Barthel sign was negative. The lumbar spine’s frontal and lateral radiographs showed the L4/5 intervertebral space narrowing. Hyperflexion and hyperextension of the lumbar spine showed that the lumbar spine was unstable. CT findings showed posterior left L4/5 disc herniation, compression of the anterior edge of the dural sac, and narrowing of the left lateral recess. MRI showed L4/5 disc herniation to the left posteriorly with nerve root compression (Fig. 1A-C). In conclusion, the patient was diagnosed with lumbar disc herniation (L4/5).

On day 7 after admission, the patient underwent PELD under local anesthesia. Immediately after the operation, the patient felt that the pain and numbness of the left lower extremity were significantly relieved. Six hours after surgery, the VAS score was 1. On the 6th day after the operation, the patient was discharged from the hospital, feeling that the symptoms were significantly relieved and the incision healed well without redness, swelling and exudation.

However, on the 10th postoperative day, the patient’s symptoms recurred with persistent dull low back pain, which aggravated after getting out of bed and was relieved by bed rest. On postoperative day 21, the patient was readmitted. Physical examination revealed lameness with limited lumbar flexion and extension. The patient presented with tenderness and percussion pain in the spinous process and between the spinous processes of the lower lumbar spine but no radiating pain. VAS score of 5 points. The skin sensation, muscle strength and muscle tone, knee tendon reflexes, and Achilles tendon reflexes of both lower extremities were normal. The straight leg raising test was negative. Further, contrast-enhanced MRI results showed that the L4/5 intervertebral disc and the left intervertebral foramen had increased signal intensity in the T1-weighted and T2-weighted phases. The edge of the enhanced scan was significantly enhanced, showing an incomplete cyst wall (Fig. 1 B, D). The patient’s body temperature was average on admission, and the ESR, CRP, and other test indicators were normal. Careful consideration was diagnosed as PDP after PELD.

**Figure 2.**
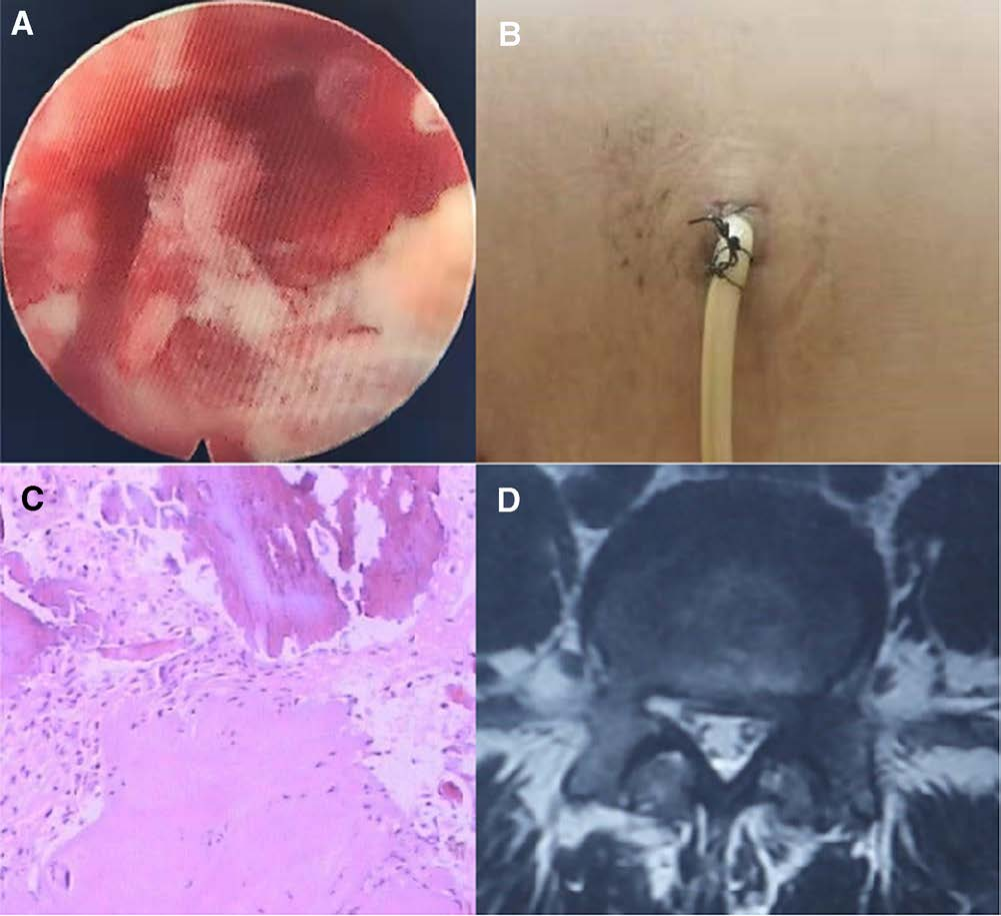
(A) Intermediate granulation tissue formation in Case 1 PELD cystectomy. (B) Placement of a 3-lumen drainage catheter after the second PELD. (C) HE staining of the surgically removed cyst showed edema of fibrous connective tissue with inflammatory cell infiltration (200× magnification). (D) Three-month follow-up MRI results showed that the strip-like enhanced signal at the original surgical site disappeared on T2-weighted images.

PELD has performed again on the second day after admission. Take the initial surgical incision as the needle entry point, puncture the needle to the L4/5 intervertebral foramen, and aspirate 3 ml of light red liquid with a syringe for bacterial culture. During the operation, it was found that there was blood oozing from part of the bone surface at the foramen formation at the site of the initial procedure. At the same time, the surrounding soft tissue was edematous, a fibrous envelope was formed, and local granulation tissue was formed (Fig. 2A). Intraoperative radiofrequency hemostasis was performed, and part of the granulation tissue was removed for pathological examination. The L4/5 intervertebral space was explored through the endoscope, and the intervertebral disc was radiofrequency ablated. It was found that there was no compression of the nerve root, and there was no apparent bleeding in the surrounding soft tissue and bone. An indwelling drainage tube was placed, and the operation was terminated (Fig. 2b). The patient’s pain symptoms were significantly relieved on a postoperative day, and the VAS score was 3 points. Sufficient drainage was performed after the operation, the drainage tube was removed on the 7th day, and about 50 ml of pale yellow liquid was drained. Drainage cell culture showed no apparent bacterial growth. Pathological findings suggested edema of fibrous connective tissue with acute and chronic inflammatory cell infiltration (Fig. 2C). The patient’s pain symptoms continued during the drainage process but gradually decreased. When the drainage tube was removed, the VAS score was 1 point. At a 3-month follow-up, the MRI results showed that the enhanced signal in the original surgical area had disappeared (Fig. 2D).

**Figure 3.**
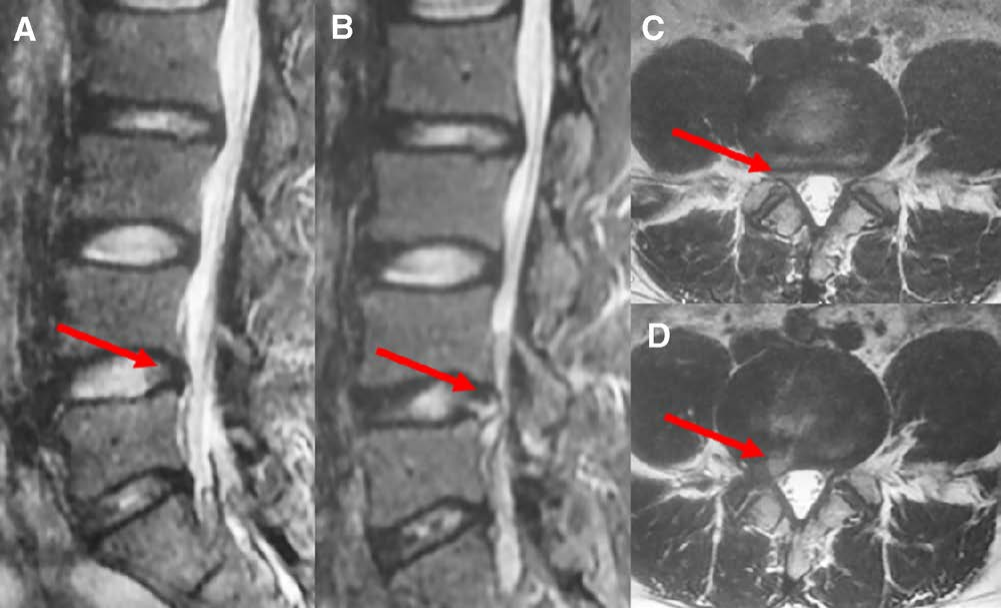
MRI images of Case 2 at the first and second admissions. (A, C) MRI of case 2 on the first admission showed that the L4/5 intervertebral disc herniated posteriorly to the right, compressing the dural sac. (A) T2-weighted sagittal image. (C) T2-weighted cross-sectional image. (B, D) MRI of case 2 on the second admission showed an enhanced signal in T2-weighted images at the L4/5 level. (B) T2-weighted sagittal image. (D) T2-weighted cross-sectional image.

Case 2: A 34-year-old man presented with low back pain for 3 months and right lower limb pain for 2 months. Physical examination after admission revealed lameness with limited lumbar flexion and extension. There was an intense lumbar spinous process, interspinous tenderness, and percussion pain, but no radiation. VAS score of 5 points. Skin sensation, muscle strength and muscle tone of both lower extremities were normal. The proper straight leg raise test at 50° was positive, but bilateral Babinski sign was negative. Both knee and Achilles tendon reflexes were normal. The lumbar spine X-ray results showed that the L4/5 intervertebral space was narrowed, and the lumbar spine hyperflexion and hyperextension X-ray results showed no lumbar instability. In addition, CT results suggested that the L4/5 intervertebral disc herniated to the right and posterior. MRI results showed that the L4/5 intervertebral disc herniated posteriorly to the right, with nerve root compression (Fig. 3A-C). In conclusion, the diagnosis was lumbar disc herniation (L4/5).

On the 2nd day after admission, the patient underwent PELD under local anesthesia, and the postoperative symptoms were significantly relieved. Six hours after surgery, the VAS score was 1. On postoperative day 4, the patient was discharged. The patient’s symptoms were relieved considerably compared with preoperative, and the incision healed well without redness, swelling and exudation.

One month after the operation, the patient had recurrent low back pain and right lower extremity pain after an accidental sprain. The results of the MRI showed no obvious abnormality in the L4/5 level, so the patient was treated with oral analgesics. Four months after the operation, the patient’s pain symptoms persisted. Another MRI examination showed that the cystic shadow in the original operation area changed into a reduced signal on the T1-weighted phase and an enhanced signal on the T2-weighted imaging and communicated with the intervertebral disc (Fig. 3B, D). The patient refused surgery and continued conservative treatment. Six months after the operation, the patient was rehospitalised with low back pain and pain in the right lower extremity. Physical examination showed limited flexion and extension of the lumbar spine, accompanied by tenderness and percussion pain in the spinous process and interspinous process of the lower lumbar spine, with a VAS score of 7. Skin sensation, muscle strength, muscle tone, bilateral knee tendon reflexes, and Achilles tendon reflexes were normal in both lower extremities. The bilateral straight leg raising test was negative. MRI examination after admission showed that the pseudocyst still existed, and there was no significant change from the previous examination. The patient’s admission body temperature was average, and CRP, ESR, and blood routine were normal.

**Figure 4.**
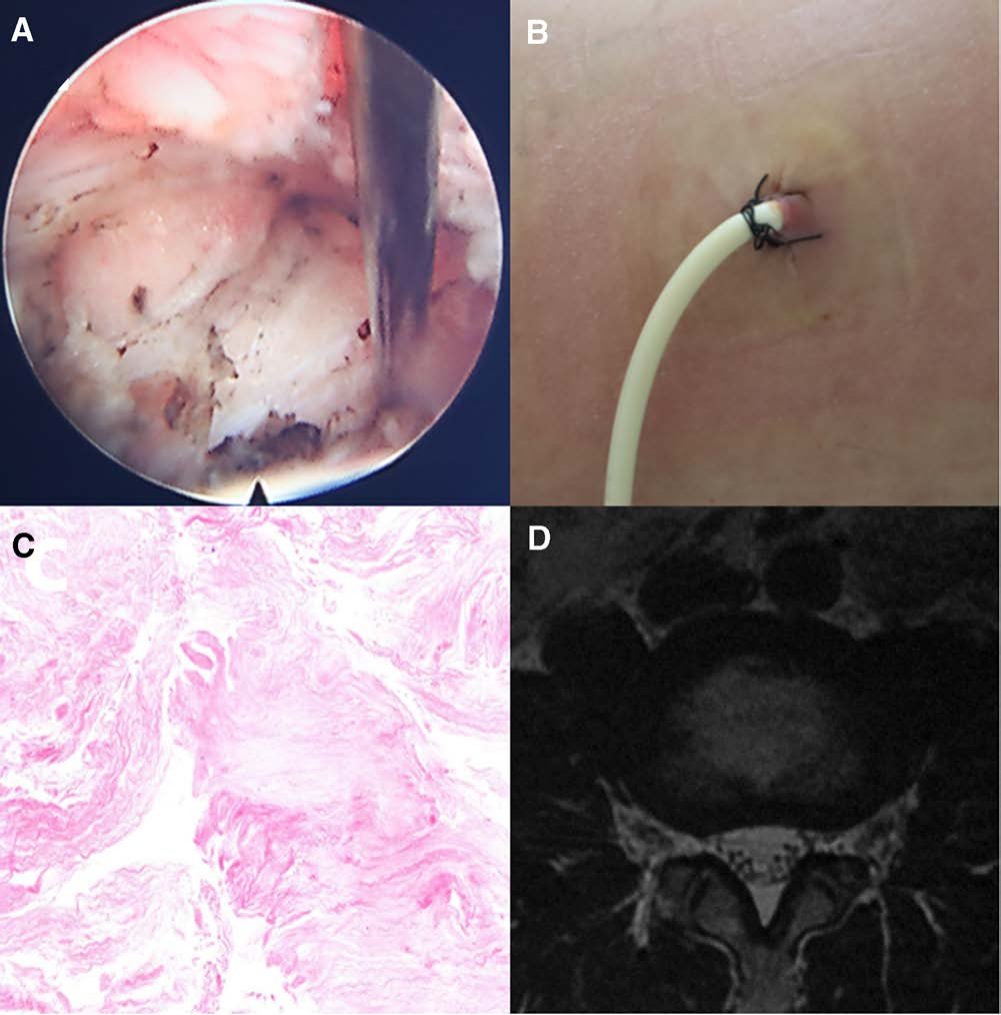
(A) A soft cystic bulge was observed during the second PELD cystectomy in case 2. (B) Place a 3-lumen drainage catheter after the second PELD. (C) HE staining of the surgically removed cyst showed degenerative nucleus pulposus tissue and hyaline change of local fibrous tissue (200× magnification). (D) Three-month follow-up MRI results showed that the enhanced signal at the original surgical site disappeared on T2-weighted images.

PELD has performed again on the 3rd day after admission. There was a cystic bulge about 0.3*0.5cm in size on the caudal side of the L4/5 intervertebral disc during the operation. Probing revealed a soft texture with fluid tissue exudation and a pale yellow cyst wall (Fig. 4A). The cyst wall was completely removed and sent for pathology. The procedure was concluded after the indwelling drainage catheter (Fig. 4B). The patient experienced pain relief immediately after the operation and a VAS score of 4 six hours after the operation. During the drainage process, the patient’s pain symptoms gradually eased. After 4 days, the drainage catheter was removed, and a total of about 5ml of pale yellow liquid was drained. The patient was discharged on the 6th postoperative day with a VAS score of 1. Pathological findings suggested degenerated nucleus pulposus tissue with localized fibrous tissue hyaline degeneration (Fig. 4C). At a 3-month follow-up, MRI showed that the pseudocyst in the original surgical area had disappeared (Fig. 4D).

## 3. Discussion

PDP is more common in young male patients, usually occurs within a short period after surgery, and often presents with clinical features of unilateral radiculopathy, including low back pain and lower extremity radiating pain. MRI is of great significance for the diagnosis of intervertebral disc pseudocysts. A cystic lesion with reduced T1W signal intensity and enhanced T2W signal that communicates with the intervertebral disc can be considered a PDP at the original surgical site.^[2,3]^ In 2009, Young^[2]^ first reported this PELD complication and named it “postoperative annular pseudocyst”. The incidence of disc pseudocyst is extremely low, Kang et al^[3]^ reported an incidence of 1%, and Ryutaro et al^[4]^ reported only 0.28%.

The mechanism of PDP formation is still controversial. One theory is that a cyst has formed before surgery, and after the disc is removed, pseudocyst forms due to fluid accumulation such as bleeding and exudation. Young et al^[2]^ believed that the herniated intervertebral disc caused an excessive inflammatory response and was surrounded by granulation tissue to form a “pseudocapsule”. The intervertebral disc is removed during the operation, and the “ pseudocapsule” may not be removed entirely. As the bleeding and exudation accumulate in the “pseudocapsule”, it gradually develops into a pseudocyst. Chung et al^[5]^ agreed with this hypothesis, arguing that mechanical compression from physical exercise would pump fluid into the “pseudo-capsule” to form pseudocysts. Qiu et al^[6]^ believed that the annulus fibrosus behind the intervertebral disc ruptured, part of the nucleus pulposus was prolapsed and deformed into a membranous structure, part of the annulus fibrosus was pushed out by the nucleus pulposus in the disc, and the 2 were connected to form a cystic cavity. Only the anteriorly prominent nucleus pulposus was removed during the operation, and the posteriorly free nucleus pulposus remained. Postoperative bleeding and exudate accumulate in the niche, forming a pseudocyst. Another theory is that no cystic cavity existed before surgery, and the surgical procedure directly led to the formation of pseudocysts. Kang et al^[3]^ believe that the construction of pseudocysts is related to the inflammation of the connective tissue at the surgical site. The radiofrequency knife burns the posterior longitudinal ligament and annulus fibrosus a lot during the operation. The high energy will aggravate the damage, cause inflammation, and lead to the formation of pseudocysts.

Interestingly, the pathogenesis and diagnosis and treatment process of the 2 cases reported in this report are different from the previous theory that the clinical symptoms of PDP are mainly caused by radiculopathy caused by cyst compression. The pseudocysts in the 2 PDP cases reported here were small and did not significantly compress the nerve roots, but the patients had significant pain symptoms. Even after the pseudocyst is removed, the pain is still noticeable. However, the pain gradually decreased during drainage. Therefore, we believe that mechanical compression does not contribute much to hurt, and pain may be more stimulated by inflammatory factors. We believe that the inflammatory response in radiofrequency burning produces an abundance of inflammatory factors. These inflammatory factors irritate surrounding tissues, especially nerve roots, leading to the recurrence of pain.

Currently, there is no clear consensus on the treatment of intervertebral disc pseudocysts. Because of the possibility of self-absorption, pseudocysts can be treated conservatively first. When conservative treatment fails, puncture aspiration and surgical treatment are commonly used. Of the 38 cases of PDP reported so far, 20 were treated conservatively, and 18 were treated surgically (Table 1). Young et al^[2]^ performed percutaneous aspiration and steroid injection for pseudocysts guided by CT, and the postoperative effect were remarkable. Kang et al^[3]^ and Chung et al^[5]^ analyzed the differences between conservative treatment and surgical efficacy. The results showed that the effects of the 2 groups were satisfactory, and only 1 case was aggravated in the conservative group of Kang et al.^[3]^ Jha et al^[7]^ reported 2 cases of young patients with mild symptoms; both received conservative treatment and recovered well. Ryutaro et al^[4]^ reported 2 cases of patients who underwent PELD revision surgery again, and postoperative pain disappeared. Qiu et al^[6]^ reported 2 cases of surgical treatment, the symptoms disappeared after surgery, and MRI results showed that the cyst disappeared. Wu et al^[8]^ reported 3 cases of PDP, 2 cases received conservative treatment and 1 case received surgical treatment, and the patients’ symptoms were significantly relieved. In treating this group of patients, we demonstrate a new strategy, PELD combined with indwelling drainage. The results showed that adequate indwelling drainage promoted the gradual relief and rapid disappearance of the patient’s symptoms.

**Table1:** Literature review of postoperative discal pseudocyst.

Author	Number of cases	Mean time to show PDP on postoperative MRI (days)	Symptoms of PDP	Treatment	Outcomes
Young et al (2016)^[1]^.	2	405	One patient had low back pain and radiative pain in the lower extremities, and the other had pain only in the lower extremities	Puncture aspiration + steroid injection	Significant relief of symptoms
Kang et al (2011)^[4]^.	15	54	Low back pain with radiating pain in the lower extremities	5 cases were treated with surgery, 10 cases were treated conservatively	Symptoms alleviated, MRI examination of conservative group 1 case of cyst enlargement
Chung et al (2012)^[5]^.	12	31.2	Radiculopathy	6 cases were treated conservatively, 6 cases were treated surgically	Symptoms disappeared, MRI examination of the cysts was significantly reduced or disappeared
Jha et al (2016)^[7]^.	2	45	Hip pain	Conservative treatment	Symptoms disappeared, 1 case of cyst disappeared completely, 1 case of cyst was significantly reduced
Ryutaro et al (2017)^[3]^.	2	50	Lower extremity pain	PELD	Symptoms disappeared
Qiu et al (2016)^[6]^.	2	45	One patient had low back pain and radiative pain in the lower extremities, and the other had pain only in the lower extremities	Operation treatment	The symptoms disappeared and the cysts disappeared on MRI
Wu et al (2019)^[8]^.	3	50	Two patients had low back pain and radiating pain in lower limbs, and 1 patient was asymptomatic	2 cases were treated conservatively,	Symptoms are relieved and the cysts disappeared on MRI
Our group	2	70.5	One patient had only low back pain, and 1 patient had low back pain with radiating pain in lower limbs	PELD and drainage	The symptoms disappeared and the cysts disappeared on MRI

This table displays literature data of postoperative lumbar disc herniation complicated with intervertebral disc pseudocyst.

PDP = postoperative discal pseudocyst, PELD = percutaneous endoscopic lumbar discectomy, MRI = magnetic resonance imaging.

In addition to revision surgery, conservative treatment is possible when patients present with complete asymptomatic or mild tolerable symptoms. However, Chung et al^[5]^ reported that the interval for self-regression of pseudocysts varied widely, ranging from 20–225 days, with an average interval of 77.8 days, and symptom relief was closely related to cyst absorption. Therefore, surgical treatment should be performed in times when the conservative treatment is ineffective or the pain resolution interval is more concerned. At the same time, we strongly recommend routine indwelling drainage during surgery to reduce the stimulation of nerve roots by local inflammatory factors and relieve radiculopathy. At the same time, we also put forward suggestions on how to prevent the formation of pseudocysts, as follows: (1) Complete hemostasis during surgery and reduce coagulation and burning; (2) Absolute bed rest on the first day after surgery; (3) Wear waist circumference for 2–3 months after surgery active, moderate physical exercise.

## 4. Conclusion

Through the intraoperative findings and postoperative management of 2 cases, we propose that the possible mechanism of PDP formation is the use of large-scale radiofrequency cautery of the surgical area to trigger a solid local inflammatory response and the cause of radiculopathy is more from the Stimulation of inflammatory factors rather than compression. For patients with ineffective conservative treatment of pseudocysts, PELD revision surgery combined with indwelling drainage is a feasible and effective new strategy for PDP treatment.

## Author contributions

ZQ.C and XC.Y. designed this study. S.W. and Y.Y. were responsible for gathering and writing the manuscript. ZQ.C and XC.Y. provided the valuable case, performed the operation, and contributed to revising the manuscript for important intellectual content. The final version of the text has been reviewed and approved by all authors.

## Acknowledgements

There was no funding for this study. The authors would like to thank Jianhua Li and Yun Bai for editing the English text for a draft of this manuscript. We thank Professor Jingming Wang for critically reviewing the manuscript.
